# Einfluss von Achsdeformitäten und deren Korrektur auf die Arthroseentstehung und -progression

**DOI:** 10.1007/s00132-021-04103-x

**Published:** 2021-04-12

**Authors:** Florian B. Imhoff, Sandro F. Fucentese, Jörg Harrer, Thomas Tischer

**Affiliations:** 1 Orthopädie, Universitätsklinik Balgrist, Forchstrasse 340, 8008 Zürich, Schweiz; 2 Abteilung für Orthopädie und Unfallchirurgie, Helmut-G.-Walther-Klinikum, Lichtenfels, Deutschland; 3 Sektion Sportorthopädie, Orthopädische Klinik und Poliklinik, Universitätsmedizin Rostock, Rostock, Deutschland

**Keywords:** Kniegelenk, Bein, Bänder, Osteotomie, Gonarthrose, Knee joint, Leg, Ligaments, Osteotomy, Osteoarthrosis

## Abstract

Die Beachtung der Beinachse und die Möglichkeiten zur deren Korrektur stellen beim jungen Patienten mit Arthrose ein Grundpfeiler der Therapie dar. Die Kombination einer Gelenksverletzung und einer Achsdeviation führt unweigerlich, je nach Ausmaß und Komorbiditäten, schon nach wenigen Jahren zur fortschreitenden Gonarthrose. Neben der genauen Deformitätenanalyse zur Osteotomieplanung, gilt es, Normbereiche der entsprechenden Winkel zu kennen und eine Zielgröße zur Achskorrektur festzulegen. Aus dem Repertoire der verschiedenen kniegelenksnahen Osteotomien sind dann die Nebeneffekte in Bezug auf patellofemorales Maltracking, ligamentäres Balancing und die Beinlänge abzuschätzen. Gerade im Hinblick auf mögliche (und wahrscheinliche) Folgeoperationen beim jungen Patienten müssen neue knöcherne Deformitäten oder ligamentäre Insuffizienzen, welche potenziell bei Überkorrektur entstehen, unbedingt vermieden werden.

## Abstract (en)

A cornerstone in the treatment of osteoarthritis in young patients is the evaluation and correction of the leg axis. The combination of a joint injury (meniscus, cartilage, ligament) and an axis deviation inevitably, depending on its extent and the patient’s comorbidities such as obesity, leads to progressive osteoarthritis of the knee after a few years. In addition to the precise deformity analysis for osteotomy planning, it is important to know the normal ranges of the corresponding angles and to define a target value for axis correction. Reflecting the repertoire of different osteotomy options around the knee (open vs. closed, tibial vs. femoral, medial vs. lateral), the side effects in relation to patellofemoral maltracking, ligamentary balancing and leg length should then be assessed. Especially with regard to possible (and probable) prosthetic operations at some time in the future of young patients, new bony deformities or ligamentous insufficiencies, which potentially arise from overcorrection, must be avoided.

Die Gonarthrose des jungen Patienten stellt nach wie vor ein schwerwiegendes Problem dar. Die endoprothetische Versorgung ist aufgrund höherer Revisionsraten und eingeschränkter Funktion hier nur bedingt geeignet. Daher ist dem Gelenkerhalt höchste Aufmerksamkeit zu schenken. Die Beachtung der Beinachse und ggf. auch die Korrektur ist ein wichtiger Pfeiler der Therapie. Neben der Auswirkung der Beinachse auf die Gelenkbelastung kam in den letzten Jahren auch immer mehr die Bedeutung der Beinachse für die Gelenkstabilität in den Fokus. Das Ziel dieses Reviews ist es, einen Überblick über den Einfluss von kniegelenksnahen Achsdeformitäten und deren Korrektur auf die Gonarthroseentstehung und -progression zu geben.

## Achsdeformitäten und deren Einfluss auf Arthrose

In der Literatur findet sich eine Vielzahl an Studien, die den grundsätzlichen Zusammenhang von frontaler Achsabweichung und späterer unikompartimenteller Gonarthrose aufzeigen. So waren in einer Untersuchung von über 5000 Knien bei bestehender radiologischer tibiofemoraler Arthrose nur 18 % neutral, jedoch 82 % mit klinischer deutlicher Achsabweichung ausgerichtet. Zudem war der Faktor Übergewicht wesentlich für das Vorhandensein (Inzidenz) einer Gonarthrose [1]. Eine weitere Studie um die Gruppe von Leesa Sharma (Chicago, USA) zeigte, dass Kniegelenke ohne Knorpelschäden infolge der chronischen Belastung durch Varusalignment ein hohes Risiko für einen späteren Knorpelverlust hatten. Nach statistischer Justierung an Alter, Geschlecht, Body-Mass-Index und lateraler Laxität hatten Varusknie eine 3,5fach erhöhte Wahrscheinlichkeit für die Entwicklung eines medialen Knorpelverlusts im Vergleich zu einem Knie ohne Varusdeformität [2]. In Bezug auf die Progression einer bestehenden unikompartimentellen Gonarthrose durch Varusalignment ist die Wahrscheinlichkeit bei einem Ausgangswert Kellgren-Lawrence (KL) Grad 2 innerhalb einer 18-monatigen Beobachtung um das 4fache erhöht; bei KL Grad 3 sogar um das 10fache [3]. Zudem wurde beschrieben, dass die Gruppe mit beidseits über 5° Achsabweichung (Varus oder Valgus) eine signifikante Verschlechterung der gemessenen Gelenksfunktion über den Beobachtungszeitraum aufzeigte [4]. Auch wenn das Durchschnittsalter in diesen Studien 64 Jahre (BMI 30,3 kg/m^2^) betrug und kein Bezug auf Vorverletzungen genommen wurde, so zeigt es zum einen die Grundlage der mechanischen Überlastung, und zum anderen, dass ein bereits (arthrotisch) verändertes Knie eine zunehmende Progression erfährt.

Bei jungen Patienten ist somit noch nicht die alleinige Achsdeviation ein Grund für die Gonarthrose, sondern vielmehr traumatische (z. B. ligamentäre Instabilitäten) oder iatrogene Veränderungen der fokalen Druckverteilung. In einer großen Follow-Up-Studie wurde festgestellt, dass die Hälfte der Knie, bei denen im jungen Erwachsenenalter eine Meniskektomie durchgeführt wurde, 21 Jahre später radiologische Zeichen der (unikompartimentellen) Arthrose aufwiesen (gegenüber 7 % in Knien ohne Meniskektomie) (Odds-Ratio für die Arthroseentwicklung: 14,0) [5]. Die Kombination einer Gelenksverletzung und einer Achsdeviation führt unweigerlich, je nach Ausmaß und Komorbiditäten wie z. B. Adipositas, schon nach wenigen Jahren zur Entwicklung der Gonarthrose beim jungen Patienten.

## Kniegelenksnahe Korrekturosteotomie

Aufgrund der oben dargestellten Ausführungen hat die mechanische Belastungsachse in der frontalen Ebene für die Diagnostik, Planung und Therapieausrichtung die entscheidende Bedeutung. Sie ist definiert als Verbindungslinie zwischen dem Zentrum des Hüftkopfes und dem Sprunggelenkszentrum. Der mechanische femorotibiale Winkel gibt vereinfacht die Gradzahl des mechanischen Varus/Valgus wieder und ist somit unabhängig von der Patientengröße ein geeigneter Vergleichsparameter.

### Planung der knöchernen Korrektur

Kniegelenksnahe Osteotomien haben das primäre Ziel einen Teil des Gelenkes zu entlasten, um der Progression einer Arthrose entgegenzuwirken. Die Umverteilung der Last wird bei medialer oder lateraler Gelenksüberlastung im frontalen Ganzbeinröntgen bestimmt und korrigiert. Auf Basis der mechanischen femorotibialen Achse, des mechanischen lateralen distalen Femurwinkels (mLDFW), des medialen proximalen Tibiawinkels (MPTW) und der Gelenklinienwinkel („joint-line conversion angle“ [JLCA]) wird eine Deformitätsanalyse dokumentiert [6]. Das Konzept, dass ein Varus immer tibial bedingt und ein Valgus femoral bedingt ist, ist heute klar verlassen [7]. Eine essenzielle präoperative Überlegung ist die postoperative Zielgröße der mechanischen Achse.

Schon 1974 empfahlen Insall et al. eine *anatomische* femorotibiale Achse von 5–10° Valgus als anzustrebende Zielachse einer kniegelenksnahen Valgisationsosteotomie, dies entspricht – je nach Anatomie – einer mechanischen Achse von ca. 1° Varus bis 4° Valgus [8]. Im Jahr 1979 beschrieben Fujisawa et al. jedoch die besten Ergebnisse nach valgisierender Umstellungsosteotomie, wenn die postoperative Traglinie bei 62 % des mediolateralen Tibiaplateaus („Fujisawa-Punkt“) lag, was einer mechanischen Achse von etwa 3° Valgus entspricht [9]. Methodisch bestehen bei beiden Arbeiten jedoch einige Schwächen.

In den letzten Jahren wurde zunehmend versucht, die Zielachse der Korrektur zu individualisieren: Jakob et al. beschrieb eine Anpassung der Zielachse je nach Restweite des medialen Gelenksspaltes im Röntgen-Ganzbein (bei 2/3 residuellem Knorpel wird auf 10–15 %, bei 1/3 auf 20–25 % und bei vollständigem Knorpelverlust auf 30–35 % geplant) [10]. Ein ähnliches Konzept wurde von Strecker et al. 2009 beschrieben, mit individueller Anpassung der geplanten Zielachse an die intraoperativ im Rahmen der simultanen Arthroskopie gefundenen Knorpelreserven (Unterschied zwischen medialer und lateraler Knorpelsituation als ∆CM): Angestrebt wurde die Verlagerung der Beinachse in die Mitte des lateralen Kompartiments (5° Valgus) bei ∆CM = IV°, auf den Fujisawa-Punkt (3,3° Valgus) bei ∆CM = III° und auf die halbe Distanz zum Fujisawa-Punkt (1,7° Valgus) bei ∆CM = II° [11].

Beide Konzepte bergen allerdings ein erhebliches Risiko der Überkorrektur! Wird bei einem quasi aufgebrauchten medialen Kompartiment sogar noch weiter als über den Fujisawa-Punkt korrigiert, kann durch das mediale Aufklappen des Gelenkspaltes bei noch relativ verlängertem Innenband bei Vollbelastung zusätzlicher Valgus entstehen und somit können Beinachsen von 8° Valgus und mehr resultieren. Ebenso droht bei lateraler ligamentärer Instabilität (Überdehnung des Außenbandes mit aufgeklapptem lateralem Kompartiment) eine valgische Überkorrektur. Daher muss bei einem erhöhten Gelenkslinienwinkel ab etwa 3–4° (JLCA) grundsätzlich eine eventuell bestehende Bandinstabilität und damit eine potenzielle Überkorrektur durch die additiven Valgusmomente nach Überschreiten des Kippunktes von Varus zu Valgus berücksichtigt und in die Planung miteinbezogen werden. Feucht et al. schlugen 2014 ein weiteres Konzept einer individualisierten Zielachse vor, bei dem die drei Zielkorridore von je 5 % je nach vorliegender Pathologie zwischen 50 und 65 % (was ca. 0–3,5° Valgus entspricht) liegen [12].

In Bezug auf eine varisierende Korrektur bei Valgusdeformität gibt es sehr wenig Evidenz. Die angestrebte postoperative Achse wird bei alleiniger Valgusfehlstellung ohne Knorpelschäden mit einem Ziel von 0°, bei valgischer Arthrose etwas medial der medialen Eminentia intercondylaris angegeben (1–1,5°). Hier sind im Grundsatz ebenfalls Überkorrekturen (> 3° Varus) wesentlich zu vermeiden und eventuell bestehende Bandinsuffizienzen analog zu beachten.

### Einstellen der Gelenklinie

Besonderes Augenmerk gilt in den letzten Jahren zudem der Wahrung oder Herstellung einer weitgehend normalen Ausrichtung der Gelenklinien und -winkel des Kniegelenkes. Es gilt bei der Korrekturplanung die Normwerte der kniegelenksnahen Winkel nach Paley [13] zu beachten und neu geschaffene Deformität (z. B. MPTW > 93°) zu vermeiden.

**Figure 1:**
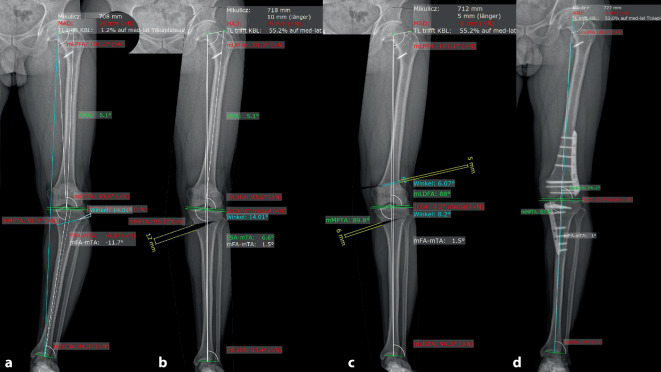

Mehrere aktuelle Studien zeigen, dass die klinischen Ergebnisse („patient related outcome scores“) und das Überleben der Osteotomie (Endpunkt = Konversion zur Prothese) abhängig vom Gelenkswinkel (JLCA) sind. Durch eine isolierte Osteotomie an Tibia oder Femur kann eine übermäßige Schrägstellung der Gelenklinie entstehen. Eine zu schräge tibiale Gelenksfläche von über 95° MPTW führt zu erhöhten Scherkräften und schlechterer Druckverteilung [14–16]. Eine neue Studie zum mittelfristigen Outcome nach HTO konnte zudem zeigen, dass ein postoperativer Winkel des Tibiaplateaus zum Boden von über > 4°, hinsichtlich klinischer Scores schlechter ist und dass sogar nachteilige Effekte ab einem Winkel von 6° bestehen [17]. In einer Analyse von über 300 Varusbeinen (mechanische Varusachse) konnte gezeigt werden, dass, wenn eine leichte tibiale Überkorrektur bis 95° akzeptiert wird, dennoch nur 57 % mit einer isolierten HTO hätten korrigiert werden können, während 33 % eine Doppelstockosteotomie bräuchten (Abb. 1; [18]).

Das Ziel der Umstellung beim jungen Patienten muss sein, die Gelenkslinie in einem „physiologischen Aspekt“ zu halten. Die Doppelstockosteotomie bietet eine gute, wenn auch chirurgisch aufwändigere Möglichkeit, gerade (physiologische) Gelenkslinien beizubehalten [19, 20]. Probleme hierbei sind einerseits die erhöhte chirurgische Schwierigkeit und die längere Operationszeit mit entsprechend erhöhten Komplikationsmöglichkeiten sowie andererseits die Mehrkosten. Allerdings ist eine femoral zuklappende Osteotomie früh belastbar und eine nur wenig öffnende tibiale Korrektur ebenfalls mit schnellerer Durchbauung und geringerem Risiko der Hinge-Fraktur zu sehen und wird daher von den Patienten sehr gut vertragen. Die Einhaltung einer „physiologischen“ Gelenkslinie und Vermeidung einer neuen Deformität ist im Hinblick auf mögliche Folgeoperationen, wie z. B. prothetische Versorgung, äußerst wichtig. Hinzu kommt die Veränderung der Beinlänge, welche je nach Osteotomieart (z. B. isolierte „open-wedge“ HTO mit 10° Korrektur) über 1 cm betragen kann, was ebenfalls durch eine Doppelstockosteotomie, wie in Abb. 1 gezeigt, ausgeglichen gehalten werden kann. Auch wenn die Beinlänge und Beckenstellung im Rahmen der Kniepathologie oft sekundär ist, so ist dieser Punkt insbesondere in die (schriftliche) Aufklärung des Patienten mit aufzunehmen!

Eine präoperative Deformitätenanalyse und entsprechende Korrekturszenarien sind extrem wichtig

**Figure 2:**
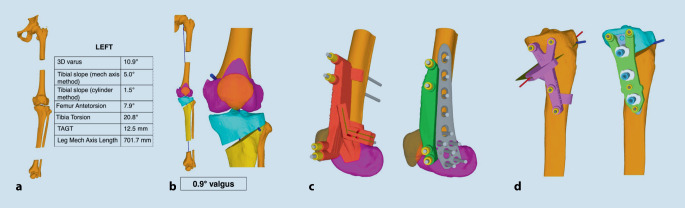

Daher sind eine präoperative Deformitätenanalyse und entsprechende Korrekturszenarien mit Zielwerten (mechanische Belastung, mechanische Achse, Grad der Arthrose, femorotibialer Gelenkswinkel, Beinlängenveränderung) extrem wichtig. Neuere Analyseverfahren wie die 3D-Analyse nach CT-Schnittbildgebung in Kombination mit einem belasteten frontalen Röntgenbild ermöglichen genaueste Winkelberechnungen, Korrekturszenarien und individuelle 3D-gedruckte Schnittblöcke (Abb. 2).

### Eigenheiten der distalen femoralen Korrektur

Im Rahmen der Deformitätenanalyse und Osteotomieplanung muss beachtet werden, dass die distale femorale Osteotomie (DFO) aus biomechanischer Sicht ein Kompartiment nur in extensionsnahen Bewegungen essenziell entlastet [21]. Entsprechend dem Extensions- und Flexionsbalancing in der Prothetik, liegt bei der DFO eine reine frontale Achskorrektur vor. Knorpelschäden im Bereich der dorsalen Kondyle werden somit durch eine tibiale Korrektur wesentlich besser in der Dynamik (Flexion über 30°) entlastet. Eine HTO wirkt sich sowohl auf den Flexions- als auch auf den Extensionsspalt aus, da sie die Tibiahöhe und theoretisch die tibiofemorale Mechanik während des gesamten Bewegungsbogens verändert [22]. Insbesondere wenn klinisch auffällt, dass ein Valgusknie auch in Flexion weiter in den Valgus abtaucht (Dysplasie der lateralen femoralen Kondyle), so ist eine tibiale („mediale closed-wedge“) varisierende Korrektur konzeptionell sinnvoller. Des Weiteren beeinflusst die DFO das patellofemorale Tracking deutlich stärker als die HTO. Dies kommt insbesondere beim Valgusknie mit lateraler Arthrose und oft simultanen patellofemoralen (PF) Beschwerden zum Tragen, da sich der Q‑Winkel effektiv verändert, während die Patellahöhe gleichbleibt [23].

### Besonderheiten des patellofemoralen Gleitlagers

Die Patellahöhe wird maßgeblich von der Osteotomietechnik (auf- oder zuklappend, supra- oder infratuberositär) im Bereich der HTO beeinflusst. Bei bestehender patellofemoraler Arthrose oder Patella baja ist die klassische aufsteigende supratuberositäre mediale „open-wedge“ HTO kritisch zu sehen, da durch weitere Distalisierung der Patella im Gleitlager mit resultierender Erhöhung des Anpressdruckes eine patellofemorale Arthrose begünstigt oder verstärkt werden kann. Daher bietet sich in solchen Fällen eine absteigende Tuberositasosteotomie an [24] oder, wenn das zu operierende Bein länger ist und keine zusätzliche Verlängerung gewünscht ist, eine früher übliche supratuberositäre laterale „closedwedge“ HTO [25].

Gerade bei der Valgusgonarthrose lässt sich, wie oben erwähnt, oft ein laterales PF-Maltracking durch die Korrektur der Achse und damit des Quadrizepsvektors zentrieren. Klinische Studien zeigen mittlerweile klar den positiven Effekt der Achskorrektur bei Patienten mit patellofemoraler Instabilität mittels Varisierung bei valgischer Beinachse [26]. Dies konnte auch in einer Kohorte mit höherem Durchschnittsalter (50±14 Jahre; Mittelwert ± Standardabweichung) und bereits degenerativ verändertem patellofemoralem Gleitlager gezeigt werden [27].

Zu einer umfassenden Deformitätenanalyse bei zusätzlicher PF-Arthrose gehört daher auch die Bestimmung der Torsion und des TT-TG(„tibial tuberosity–trochlear groove“)-Abstandes. Eine aktuelle Studie mit MRT- und CT-Datensätzen konnte einen positiven Zusammenhang von femoraler Torsion und patellofemoraler Arthrose darlegen. Eine femorale Antetorsion von über 20° zeigte höhergradige Knorpeldegeneration im Bereich der lateralen Retropatellarfläche versus medial, was in Kombination mit einem Valgusalignment noch verstärkt wurde [28]. Dahingehend kann ein vorderer Knieschmerz nach valgisierender HTO insbesondere bei Frauen mit generell etwas höherer femoralen Antetorsion hypothetisch auf eine Dekompensation des patellofemoralen Trackings hinweisen. Allerdings gibt es hierzu noch keine validen klinischen Daten.

### Osteotomie bei Bandinsuffizienzen

Achsdeformitäten haben nicht nur einen direkten Einfluss auf die Gelenkbelastung, sondern auch einen Einfluss auf die ligamentäre Belastung und Stabilität des Kniegelenkes und können damit indirekt zur Arthroseprogression beitragen^1^ [29].

In biomechanischen Studien konnte gezeigt werden, dass das koronare Alignement einen wichtigen Einfluss auf die Spannung des vorderen Kreuzbandes (VKB) besitzt [30, 31]. Ein Varus-Thrust führt zu erhöhter Belastung des VKB. Gleichzeitig führt allerdings auch ein deutlicher Valgus zu einer erhöhten Belastung des VKB. Das koronare Alignment hat zudem einen wichtigen Einfluss auf die Seitenbandstabilität und sollte zwingend bei allen Seitenbandinstabilitäten berücksichtigt und ggf. therapiert werden [32].

**Figure 3:**
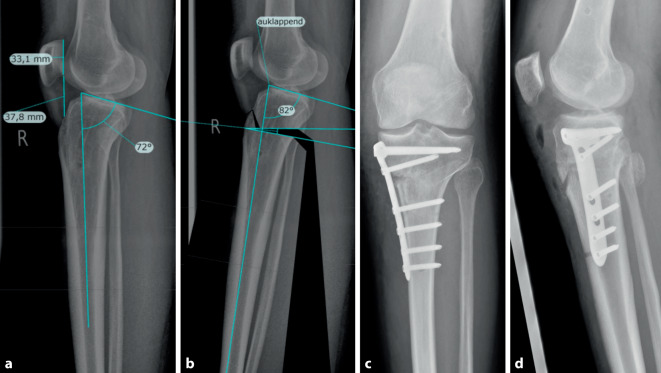

In Kadaverstudien konnte zudem gezeigt werden, dass der posteriore tibiale Slope einen wichtigen Einfluss auf die sagittale Stabilität des Kniegelenkes hat [33, 34]. Inzwischen ist eindeutig belegt, dass ein erhöhter tibialer Slope ein Risikofaktor für eine Verletzung bzw. Re-Instabilität nach VKB-Rekonstruktion ist (Abb. 3). Das gleiche gilt umgekehrt bei erniedrigtem Slope auch für Verletzungen des hinteren Kreuzbandes [35]. Zudem zeigen biomechanische Daten, dass bei einem VKB-insuffizienten Kniegelenk eine alleinige Korrektur des Varus zu einer erhöhten tibialen Translation führt und daher eine kombinierte Slopereduktion als dreidimensionale Korrekturosteotomie angestrebt werden sollte (Abb. 3 und 4; [36]).

**Figure 4:**
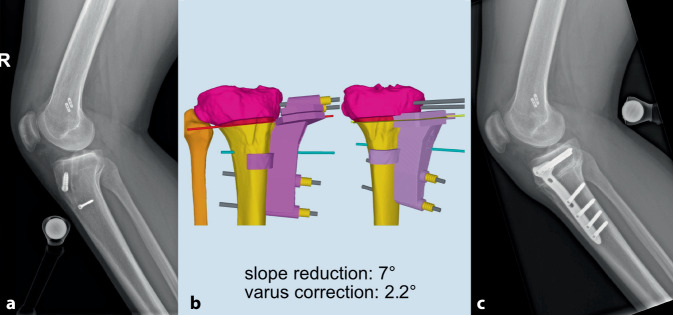

Zusammenfassend zeigt sich in den Grundlagenstudien, dass Achsdeformitäten sowohl in koronarer als auch sagittaler Ebene einen wichtigen Faktor für die Stabilität des Kniegelenkes sowie das Versagen von Bandplastiken darstellen und durch knöcherne Korrekturen wesentlich beeinflusst werden können.

## Ausmaß der Achskorrektur beim jungen Arthroseknie

Berücksichtigt man die Ergebnisse aus den biomechanischen sowie klinischen Studien empfehlen die Autoren im Konsensus folgendes Konzept:

### Ab wann ist die Indikation zur Achskorrektur beim jungen Patienten gegeben?

- symptomatische unikompartimentelle Überlastung ab 5° mechanische Achsfehlstellung
- vorhandene mediolaterale ligamentäre Insuffizienz bereits ab 3° mechanische Achsfehlstellung
- unikompartimentelle Knorpel- und/oder Meniskuschirurgie ab 2–3° mechanische Achsfehlstellung – *Beachte:* Ein Korrekturwinkel um mindestens 4° ist nötig um eine relevante Belastungsverschiebung zu erzielen [37].
- patellofemorales Maltracking und Valgusdeformität ab 3–4°
- bei Kindern mit offenen Wachstumsfugen und asymptomatischer Achsfehlstellung kann ab 5° eine Epiphysiodese erwogen werden.
- bei Erwachsenen mit asymptomatischer Achsdeviation über 8° kann bei entsprechendem Wunsch eine prophylaktische Achskorrektur erwogen werden (keine medizinische/orthopädische Indikation).

### Angestrebte mechanische Zielachse bei Varusdeformität beim jungen Patienten:

- ohne relevante Arthrose wird eine 0–0,5° Valgusachse angestrebt, entsprechend ca. 50–53 % der mediolateralen tibialen Belastung
- bei chronischer degenerativer Innenmeniskuspathologie, mittelgradiger bis hochgradiger Arthrose wird eine Zielachse von 1,5–2,5° Valgus angestrebt, entsprechend ca. 55–60 % mediolaterale tibiale Belastung – Bei geringgradiger Arthrose bzw. nicht bestehender Instabilität wird diese durch Belastung optimal bei 2° Valgus verbleiben; bei höhergradiger Arthrose mit hohem JLCA und evtl. bestehender seitlicher Instabilität ist trotz zusätzlich entstehendem Valgusmoment keine relevante Überkorrektur zu erwarten.
- tibiale Zielgelenkwinkel von über 93° MPTW gilt es zu vermeiden

### Angestrebte mechanische Zielachse bei Valgusdeformität beim jungen Patienten:

- ohne relevante Arthrose wird eine 0°-Achse angestrebt
- bei lateraler Arthrose sollte die Korrektur 1–1,5° Varus nicht übersteigen
- patellofemorales Tracking beachten

## Outcome nach Osteotomie

In einer systematischen Übersichtsarbeit von 7087 tibialen Osteotomien zeigten sich im mittleren Follow-Up von über 10 Jahren Überlebensraten nach 5, 10, 15 und 20 Jahren von 86–100 %, 64–97,6 %, 44–93,2 % bzw. 46–85,1 %. Zusammengefasst wurde das „optimale“ Korrekturergebnis mit 0,6–4° Valgus bei vorbestehenden Varuspathologien angegeben [38]. Risikofaktoren für ein Versagen waren v. a. höheres Alter, Bluthochdruck, andere Komorbiditäten und weibliches Geschlecht. Eine begleitende Knorpeltherapie kann das arthroskopische und histologische Outcome verbessern, jedoch ist bislang kein vorteilhafter Effekt auf das klinische oder radiologische Outcome belegt [39, 40]. Zudem zeigten sich neben guten Outcome-Scores wesentliche Verbesserungen im Gangbild (mechanische Tragachse und Knieadduktionsmoment) 5 Jahre nach Intervention [41].

Bezüglich der distalen femoralen Osteotomie werden ebenfalls in einzelnen Studien sehr gute Langzeitergebnisse, respektive Standzeiten bis zur operativen Konversion, angegeben: 10 Jahre (90 %), 15 Jahre (79 %), 20 Jahre (21,5 %) [42]. Eine weitere aktuelle Studie zeigt eine 89-%-Überlebensrate der DFO bei lateraler Arthrose nach 10 Jahren, wobei der präoperative Mikulicz-Punkt in Prozentwerten bei 78,7 % (SD 19,1 %) (= valgus) und postoperativ bei 35,9 % (SD 14,8 %) (= varus) lag [43].

Das Ergebnis nach HTO und zeitgleicher VKB-Ersatzbandplastik bei Patienten mit chronischer vorderer Instabilität und Varusstellung mit medialer Arthrose wurde von Marriott et al. mittels Ganganalyse und Scores aufgezeigt. Die Auswertung der präoperativen, 2‑Jahres- und 5‑Jahres-Daten zeigten eine klare Verbesserung der Gelenksbiomechanik (Gangbild) durch eine Abnahme der zuvor bestandenen Knieadduktions- und -flexionsmomente und anhaltend hohe Patientenzufriedenheit [44].

### Komplikationen nach HTO

Ein wesentliches Problem nach Umstellung ist neben der Unterkorrektur (bis zu 62 %) die Überkorrektur (bis zu 16 %) der mechanischen Tragachse [45]. Die mechanische Überkorrektur nach HTO zeigte in der Studie von Briem et al. ein deutlich schlechteres Gangbild sowie, wenig überraschend, schlechtere Outcome-Scores [46]. Insgesamt zeigten die Daten der Regressionsanalyse bei Patienten mit medialer Arthrose ein schlechteres Ergebnis entweder bei einer unzureichenden oder einer übermäßigen Korrektur der Varusdeformität [46]. Neben der störenden Ästhetik beim übertriebenen Valgusbein ist vor allem der Stress auf das mediale Seitenband hervorzuheben [47]. Diese schmerzsensible Struktur bedingt aufgrund des ständigen Zuges eine andauernde Schmerzafferenz. Zudem bedingt es eine zunehmende Insuffizienz des Innenbandes, sodass im weiteren Verlauf sogar höhergekoppelte Prothesensysteme verwendet werden müssen. Eine Patella baja sollte unbedingt vermieden werden, um nicht neue vordere Knieschmerzen zu generieren oder eine bestehende patellofemorale Chondromalazie zu aktivieren. Die distal auslaufende tibiale Osteotomie ist daher eine gute Option bei dieser Gefahr. Die besagten Probleme nach HTO können durch eine saubere präoperative Planung und entsprechend durchgeführte Chirurgie, falls möglich mit Hilfe von Navigation oder patientenspezifischen Schnittblöcken, besser kontrolliert werden.

### Konversion zur Prothetik

Trotz des aufgezeigten Zeitgewinns und der Verzögerung der Arthroseprogression beim jungen Patienten wird im Rahmen der zunehmenden Lebenserwartung eine Konversion auf einen endoprothetischen Ersatz wahrscheinlicher werden. Die aktuelle Knieendoprothetik mit einer zementierten Prothese nach HTO zeigt eine hervorragende Langzeitbeständigkeit mit einer 10-jährigen Überlebensrate ohne aseptische Lockerung von 97 % mit insgesamt zuverlässig verbesserten klinischen Scores im Langzeit-Follow-Up. Allerdings wird in diesem Beobachtungszeitraum eine Prävalenz von 4 % Narkosemobilisationen und 3 % Revision aufgrund einer ligamentären Instabilität angegeben, was nach Chambers et al. auf ein schwierigeres Balancing nach HTO zurückzuführen sein könnte [48]. Zudem ist die Infektrate von 1,4 % versus 1,0 % etwas höher, was auf die technisch anspruchsvollere Operation mit längerer Operationsdauer und höherem Blutverlust, sowie Zustand nach Voroperation zurückzuführen ist [49].

**Figure 5:**
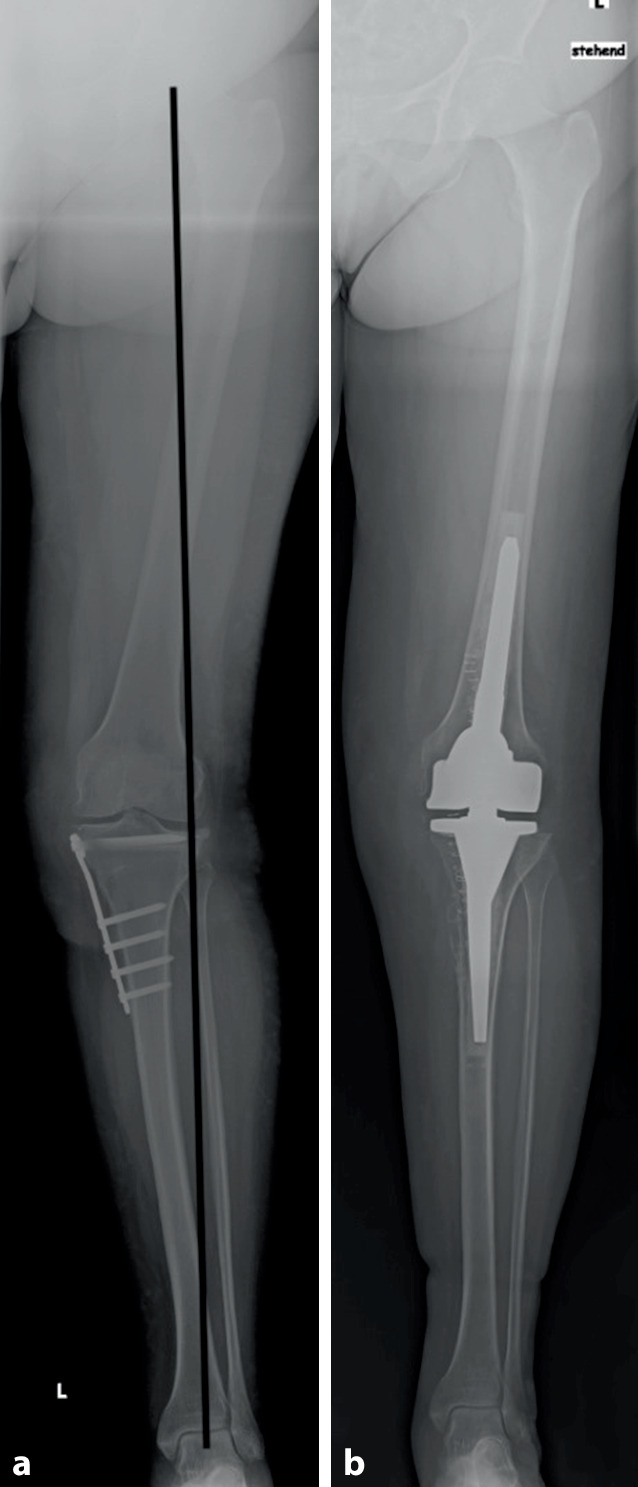

Eine (auf die Endoprothetik) vorausschauende Osteotomieplanung beinhaltet damit die klare Vermeidung von neuen Achsdeformitäten, wie oben ausführlich beschrieben. Eine laterale „closed-wedge“ HTO erschwert aufgrund des Impingements zwischen Schaft/Keil und des Missalignments der Kortikalis die Verankerung der tibialen Komponente, was historisch gesehen ein großes Problem war und zu einem gewissen Punkt den schlechten Daten nach Konversion von vor 20–30 Jahren zugrunde lag [49, 50]. Zudem können durch eine schräge Gelenklinie nach „überzogener“ Tibiakopf-Valgisationsosteotomie Balancing-Probleme entstehen, da die tibiale Resektion sehr varisch wird und hierbei Streck- und Beugespalt beeinflusst. Bei zuvor überkorrigiertem MPTW führt dies zu einem extrem schwierigen Weichteilrelease, um das Gelenk stabil zu bekommen und den Patellalauf korrekt einzustellen. Teils kann dies sogar die Implantation eines Oberflächenersatzes unmöglich und eine (teil)gekoppelte Prothese erforderlich machen (Abb. 5; [51]).

Eine bestehende HTO ist keine Kontraindikation für eine mediale unikompartimentelle Prothese, da mittelfristig gute bis sehr gute klinische Ergebnisse erzielt werden können, wenn präoperativ eine möglichst gerade (oder leicht varische) Achse besteht und postoperativ keine relevante neue Valgusachse durch die Prothese entsteht [52]. Allerdings sollte der Grund für das „Versagen“ der HTO (Unterkorrektur, ligamentäre Insuffizienz) vor einer unikompartimentellen Arthrose genauestens evaluiert werden. Die präoperative Vorbereitung beinhaltet erneut die Analyse der knöchernen Geometrie (präoperative mechanischen Achse, Angulierung/Konvergenz der Gelenkebenen = JLCA) sowie der medialen Bandlaxität und des Zustands des lateralen Kompartimentes.

Beim initialen Zugangsweg ist eine zu mediale Schnittführung zur HTO oder medialen DFO zu vermeiden, im Hinblick auf eine mögliche spätere Folgeoperation, wo die Exposition des Gelenkes dadurch deutlich erschwert sein kann. Eine nach exzessiver HTO entstandene Patella baja gilt in diesem Zusammenhang ebenfalls als technisches Problem, worauf schon primär geachtet werden sollte [53–55]. Zudem ist ein persistierender Knieschmerz in der Gruppe der Patienten nach HTO häufiger gefunden worden [56], sodass ein primärer Patellarückflächenersatz in dieser Patientenpopulation eher sinnvoll ist, um die Reoperationsrate niedriger zu halten [50]. Die Konversion von einer HTO zu einer Totalendoprothese ist keine Anfängeroperation und bedarf ebenfalls einer genauen präoperativen knöchernen und ligamentären Beurteilung.

## Fazit für die Praxis

- Die Korrektur von Achsdeformitäten ist ein essenzieller Behandlungspfeiler der Gonarthrose beim jungen Patienten.
- Der mechanisch bedingte Einfluss von Achsdeformitäten ist evident und der Progression der Erkrankung kann somit entgegengewirkt werden.
- Die operative Korrektur benötigt eine umfassende präoperative Analyse und Zielgröße, sodass auch für zukünftige operative Eingriffe (ligamentäre Rekonstruktion, prothetische Konversion) möglichst optimale Bedingungen bestehen bleiben.

## Abkürzungen

∆CM: Unterschied zwischen medialer und lateraler Knorpelsituation
BMI: Body-Mass-Index
DFO: Distale femorale Osteotomie
HTO: Hohe tibiale Osteotomie
JLCA: „Joint-line conversion angle“
mLDFW: Mechanischer lateraler distaler Femurwinkel
MPTW: Medialer proximaler Tibiawinkel
PF: Patellofemoral
TT-TG: „Tibial tuberosity–trochlear groove“
VKB: Vorderes Kreuzband

## Footnotes

1 siehe hierzu auch Artikel „Instabilitätsarthrose: Stellenwert der Bandrekonstruktion bei bestehender Arthrose“ in diesem Heft.
